# Keratitis in patients with corneal foreign bodies: a cross-sectional study in Cali, Colombia

**DOI:** 10.5935/0004-2749.2022-0257

**Published:** 2023-09-27

**Authors:** Diego Andres Guarin, Francisco Javier Bonilla-Escobar, Omar Salamanca, Gerson López Moreno, Alexander M. Martínez-Blanco

**Affiliations:** 1 Grupo de Investigación en Visión y Salud Ocular, Department of Ophthalmology, Universidad del Valle, Cali, Valle del Cauca, Colombia; 2 Department of Ophthalmology, School of Medicine, University of Pittsburgh, USA; 3 Orbis International, NY, USA; 4 Clínica Oftalmológica de Cali, Valle del Cauca, Colombia; 5 Pontificia Universidad Javeriana, Colombia; 6 Department of Ophthalmology, Clínica Imbanaco Grupo Quirón Salud, Cali, Colombia

**Keywords:** Eye foreign bodies, Corneal injuries, Keratitis/epidemiology, Cross-sectional studies, Colombia, Corpos estranhos no olho, Lesões da córnea, Ceratite/epidemiologia, Estudos transversais, Colômbia

## Abstract

**Purposes:**

To describe the clinical characteristics and factors associated with keratitis in patients with corneal foreign bodies in Colombia.

**Methods:**

This cross-sectional study was based on a clinical records review of patients who had corneal foreign bodies and were admitted to the emergency department between June 2018 and June 2019 in Cali, Colombia. The primary outcome was the presence of keratitis diagnosed based on clinical criteria. Univariate and multivariate logistic regression models were used to identify associated factors.

**Results:**

A total of 381 corneal foreign bodies in 372 patients were analyzed (median age, 40.0; interquartile range, 29.0-53.0 years; male, 94.7% (n=352). Ninety-five patients developed keratitis (24.9%, 95% confidence interval [CI] 20.8%-29.5%). In the multivariate analysis, age 30 years (odds ratio [OR] 2.15, 95% CI 1.06-4.36), finding of aqueous flare (OR 2.81, 95% CI 1.39-5.66]), and a foreign body in the peripheral cornea (OR 2.05, 95% CI 1.19-3.50] were associated with an increased risk for keratitis. Sex, time between injury and admission, and corneal edema were not related to keratitis (p>0.05).

**Conclusion:**

In Cali, Colombia, a high proportion of keratitis was reported in patients with corneal foreign body. Age, an aqueous flare, and a foreign body in the peripheral cornea were the factors associated with keratitis.

## Abstract (pt)

**Objetivo:**

Descrever as características clínicas e os fatores associados à presença de ceratite em pacientes com corpos estranhos na córnea em uma população colombiana.

**Métodos:**

Trata-se de um estudo transversal baseado na revisão dos registros clínicos de pacientes com corpos estranhos na córnea admitidos em um departamento de emergência em Cali, Colômbia, entre junho de 2018 e junho de 2019. O desfecho primário foi a presença de ceratite diagnosticada através de critérios clínicos. Foram utilizados modelos de regressão logística univariada e multivariada para identificar os fatores associados.

**Resultado:**

Neste estudo, foi analisado um total de 381 corpos estranhos na córnea em 372 pacientes (idade média: 40,0 anos, intervalo interquartil: 29,0-53,0; sexo masculino: 94,7% [352 casos]). Noventa e cinco casos desenvolveram ceratite (24,9%, intervalo de confiança de 95% — IC 95%: 20,8%-29,5%). Na análise multivariada, para idade ≤30 anos (razão de chances — RC: 2,15, IC 95%: 1,06-4,36), o achado de *flare* aquoso (RC: 2,81, IC 95%: 1,39-5,66]) e a presença de corpo estranho na periferia da córnea (RC: 2,05, IC 95%: 1,19-3,50) foram associados a um risco aumentado de ceratite. Sexo, tempo entre a lesão e a internação, e edema da córnea não foram relacionados à ceratite (p>0,05).

**Conclusão:**

Há uma proporção elevada de ceratite em casos de corpos estranhos na córnea em Cali, Colômbia. Os três fatores associados à ceratite foram a idade, o achado de *flare* aquoso e a presença de corpo estranho na periferia da córnea.

## INTRODUCTION

Corneal foreign body (CFB) is one of the reasons for visiting the ophthalmology emergency department worldwide, representing 6.2%-40.0% of all ocular consultations^(1,2,3,4)^. CFBs are most common in men aged 25-34 years and often occur as an occupational-related injury attributed to not wearing eye protection^(5,6)^. The initial treatment consists of foreign body removal and administration of antibiotics and analgesics to prevent infections, promote corneal epithelization, avoid corneal opacities, and control pain^(7)^.

Although many patients have a favorable recovery, a CFB can cause inflammation and infection even after the foreign body was removed^(8)^. Keratitis is a potential complication of CFB injury and is considered a sight-threatening event, contributing to the world’s high prevalence of corneal blindness^(9)^. Trauma favors bacterial colonization, and positive conjunctival swabs have been documented in 20% of patients with CFBs^(10,11)^. Although traumatic etiology represents at least a third of keratitis cases^(8)^, studies on the epidemiology of CFB and keratitis are limited, especially in low-, and middle-income countries^(12)^.

Therefore, this study aimed to describe the clinical characteristics and factors associated with the presence of keratitis in patients with CFBs in a Colombian population.

## METHODS

This cross-sectional study was based on clinical records review of patients who had CFBs and admitted to the emergency department between June 2018 and June 2019 in Cali, Colombia. This study was performed in a high-complexity center that provides health services to people with any type of healthcare insurance from the southwestern region of Colombia. Between 2018 and 2019, 52,000 emergencies were served, and of these, 1700 (3.3%) visits were related to ocular problems.

This study was approved by the Institutional Review Board (Approval codes 020-2020 and 063-020) and conducted in accordance with the Declaration of Helsinki.

All consecutive patients diagnosed with CFBs were identified through the institutional ophthalmologic emergency registry. Patients with code T150 according to the International Classification of Diseases (ICD-10), Tenth Revision, were included. Those who were using homeopathic eye drops on admission or had a history of corneal dystrophies were excluded.

### Data collection

Data were obtained from the institutional ophthalmologic emergency registry. All data were also validated with the patient’s medical records. Demographic and clinical characteristics were collected, including the duration from injury to ophthalmology consultation, physical examination findings, and CFB location (based on the visual axis: 1-2 mm at the central zone; paracentral, 3-4 mm from the central zone; periphery, ≥7 mm) with the slit lamp. The primary outcome was the presence of keratitis, and eye diagnosis was based on ICD-10 H16 code and in ophthalmology discharge notes.

### Statistical analysis

Continuous variables were reported as the median and interquartile range (IQR) because they were not normally distributed. Categorical variables were summarized as frequencies and percentages. Normality was tested using the Shapiro-Wilk test.

The percentage of keratitis was estimated using Wilson approximation with its respective 95% confidence interval (CI). The Mann-Whitney nonparametric test was used to compare continuous variables between patients with and without keratitis. The Chi^2^-test and Fisher exact tests were used to analyze categorical variables. Associations with keratitis were identified using univariate and multivariate logistic regression models and reported with the corresponding odds ratios (OR) and 95% CI. A backward elimination algorithm was applied to construct the multivariate model with the variables that were significant at the 0.20 level in the univariate models. The goodness of fit of the final model was tested using the Pearson goodness-of-fit test. All statistical analyses were performed in Stata version 16.0 (StataCorp, College, Station, TX), and p-values <0.05 (two-sided) were considered statistically significant. In the univariate and multivariate analyses, missing data were handled using a complete case approach.

## RESULTS

A total of 419 CFBs were identified during the study period, and 34 cases with a history of using homeopathic eye drops or corneal dystrophies and four cases with an inconclusive diagnosis of keratitis were excluded. Ultimately, 381 CFBs in 372 patients, with a median age of 40.0 (IQR: 29.0-53.0) years, were studied. Of these patients, 94.7% (352) were male, and 2.4% (9) had foreign bodies in both eyes. The median number of days between injury and admission to the emergency department was 1 day (IQR: 1-3 days). Most foreign bodies were located in the paracentral (41.2%) and peripheral (41.2) regions of the cornea. Aqueous flare and corneal edema were observed in 16.8% (44) and 7.3% (19) of patients with available data, respectively (Table 1).

**Table 1:** Comparison of the sociodemographic and clinical characteristics of patients with and without keratitis

Characteristics	Total (n=381)	Keratitis (n=95)	Non-keratitis (n=286)	p-value
Age, yr				
Median (IQR)	40.0 (29.0-53.0)	37.0 (26.0-47.0)	41.0 (29.7-55.0)	0.033
≤30	110 (28.9)	33 (34.7)	77 (26.9)	
31-40	84 (22.0)	22 (23.2)	62 (21.7)	
41-50	70 (18.4)	19 (20.0)	51 (17.8)	
51+	117 (30.7)	21 (22.1)	96 (33.6)	
Sex, n (%)				
Male	361 (94.7)	89 (93.7)	272 (95.1)	0.591
Population, n (%)				
No.	326	79	238	0.340
Rural	14 (4.3)	5 (6.3)	9 (3.6)	
Urban	312 (95.7)	74 (93.7)	238 (96.4)	
Eye, n (%)				
Right	197 (51.7)	46 (48.4)	151 (52.8)	0.479
Left	184 (48.3)	49 (51.6)	135 (47.2)	
Time between injury and admission, days				
No.	371	92	279	0.327
Median (IQR)	1 (1-3)	1 (1-3)	1 (1-3)	
3 days, n (%)	97 (26.1)	29 (31.5)	68 (24.4)	
Signs				
Aqueous flare, No.	262	91	171	
n (%)	44 (16.8)	24 (26.4)	20 (11.7)	0.002
Corneal edema, No.	261	90	171	
N (%)	19 (7.3)	10 (11.1)	9 (5.3)	0.084
Location CFB, n (%)				
Visual axis	47 (12.3)	8 (8.4)	39 (13.6)	0.180
Paracentral	157 (41.2)	34 (35.8)	123 (43.0)	0.216
Periphery	157 (41.2)	48 (50.5)	109 (38.1)	0.033

IQR= interquartile range; No.= number of cases with available data; Yr= years.

Of the 381 eyes with CFBs, 95 (24.9%, 95% CI 20.8%-29.5%) were clinically diagnosed with keratitis. The keratitis group was younger and had a higher proportion of aqueous flare and corneal edema findings than the non-keratitis group (p<0.05). In addition, foreign bodies in eyes with keratitis were commonly located in the peripheral cornea (50.5% vs. 38.1%). The time from injury to emergency room admission was comparable between patients with or without keratitis (Table 1). However, a higher percentage of patients with a delay of ≥3 days was reported in the keratitis group (31.5% vs. 24.4%).

**Figure 1:**
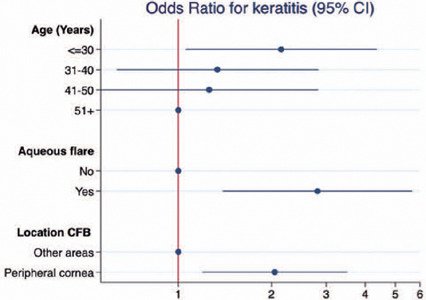
Factors associated with keratitis in patients with corneal foreign body (CFB).

In the multivariate analysis, younger age (30 years), an aqueous flare, and a foreign body located in the peripheral cornea were associated with an increased risk of keratitis (Table 2, Figure 1).

**Table 2:** Univariate and multivariate logistic regression analyses for keratitis in patients with CFBs

Characteristics	Univariate OR (95% CI)	p-value	Multivariate OR (95%CI)^a^	p-value
Age, yr				
≤30	1.96 (1.05-3.66)	0.035	2.15 (1.06-4.36)	0.035
31-40	1.62 (0.82-3.19)	0.162	1.34 (0.63-2.82)	0.443
41-50	1.70 (0.84-3.46)	0.140	1.26 (0.56-2.82)	0.572
51+	Reference	-	Reference	
Sex, Male	0.76 (0.28-2.05)	0.592	-	-
Population, n (%)				
Urban	Reference		-	-
Rural	1.79 (0.58-5.50)	0.311		
Time between injury and admission, days	1.04 (0.96-1.12)	0.305	-	-
Signs				
Aqueous flare	2.70 (1.40-5.23)	0.003	2.81 (1.39-5.66)	0.004
Edema Corneal	2.25 (0.88-5.76)	0.091	-	
Location CFB				
Visual axis	0.58 (0.26-1.29)	0.185	-	
Paracentral	0.74 (0.46-1.19)	0.216	-	
Periphery	1.66 (1.04-2.65)	0.034	2.05 (1.19-3.50)	0.009

CI= confidence interval; OR= odds ratio. ^a^= Regression estimated with 261 observations.

## DISCUSSION

This study reveals that younger age (30 years), an aqueous flare, and a foreign body in the peripheral region of the cornea increase the risk of developing keratitis in patients with CFBs. In Cali, Colombia, approximately 1 in 4 patients with CFBs developed keratitis, which is comparable to that reported in France (27.7%)^(13)^. However, this complication rate was higher than that estimated in patients with CFBs from the United States (4.0%)^(5)^ and Canada (1.7%)^(14)^, whereas our keratitis rate was lower than previously reported in Indonesia (33.3%)^(8)^. This study was conducted in a public institution, and probably, most of the patients belong to vulnerable population groups with low income and perhaps less access to a healthy environment (e.g., quality water), which may explain the high proportion found in this cohort.

Although several reports have described the epidemiology of keratitis, a few studies have examined the frequency and risk factors in patients with CFBs; therefore, these patients have been analyzed within the group with traumatic keratitis^(12)^. Several organisms can cause corneal infections, including bacteria, viruses, fungi, and protozoa. The types of pathogens found in patients with traumatic keratitis were mainly related to the geographical region and occupation; more cases of fungal keratitis were described in low-income countries (e.g., Asian, or African countries) and more cases of bacterial keratitis in high-income countries (e.g., United States or European countries)^(12)^.

Ocular trauma, including foreign body injuries, is a strongly associated factor that increases the risk of keratitis. For example, in a case-control study from Uganda, patients with and without keratitis reported a history of ocular trauma in 29% and 0% of patients, respectively^(15)^. This excess risk in patients with CFBs has been attributed mainly to the presence of contaminated foreign bodies. According to DeBroff et al. and Macedo et al., foreign bodies with positive cultures have been reported in 14.3% and 32.7%, respectively^(10,16)^. *Staphylococcus aureus, S. epidermidis, Streptococcus pneumonia,* and *Pseudomonas aeruginosa* are the most common causative pathogens in patients with CFB^(12,17,18)^.

Young adults are at higher risk for CFB because they tend to suffer more work-related eye injuries due to the lack of eye protection. This may explain why decreasing age can predict an increased risk of keratitis in this study. In addition, younger adults are less adherent to medical recommendations and use of eye drops^(19)^, creating an optimal environmental condition for pathogens to grow.

In this study, a higher keratitis risk was identified when the foreign body was located in the peripheral zone of the cornea, which is thicker, and has fewer sensitive receptors than the central zone^(20)^. Consequently, foreign bodies embedded in this zone likely generate less foreign body sensation and other symptoms, which delay their identification, and the risk of keratitis development is increased if the object is infected. In this corneal zone, ocular manifestations such as ulcers or corneal infiltrates have been frequently reported in patients with autoimmune or other systemic diseases, which are attributed to a higher density of inflammatory cells (e.g., leukocytes, lymphocytes, and eosinophils) that increase the susceptibility to immune reactions^(21,22)^.

On the contrary, an aqueous flare is more commonly found in eyes with keratitis during an ophthalmologic examination. This sign indicates initial damage to the corneal epithelium and is induced as an innate immune response against the foreign body and keratitis process^(22)^. Bacteria can generate an inflammatory process by combining with the pathogen’s molecular patterns and toll-like receptors present on the corneal surface, leading to increased production of inflammatory cytokines and ongoing aqueous flare^(23)^. Therefore, an aqueous flare can alert the need for close follow-up and consider the use of prophylaxis with antibiotics after CFB removal.

In the treatment of CFBs, although it could be argued based on clinical experience that a longer duration from injury to removal could be associated with increased keratitis risk, the results do not support this assumption. However, most patients with CFBs are involved in occupational injuries, which could favor immediate access to healthcare. Moreover, the time interval between injury and admission was measured in days, not in hours, which did not allow us to assess whether the risk of keratitis could vary according to waiting times on the same day. In general, regardless of the type of material, size, location, or depth of the foreign body, prompt removal is recommended to reduce the occurrence of potential complications, such as keratitis, edema, scarring, or reduced vision. In addition, a longer duration of CFB implantation may make removal more difficult^(24,25)^.

This study has some limitations. First, the retrospective design did not allow the collection of all variables of interest, such as foreign body materials or microbiological characteristics of keratitis. In addition, data on some relevant variables were missing, which decreased the number of observations to be analyzed and the statistical power. Second, all cases were identified at a single institution, which limits the generalizability of our results. Despite these limitations, the findings represent a starting point to reach a deeper understanding of the development of keratitis in patients with CFBs.

In conclusion, the proportion of keratitis in patients with CFB is high in Cali, Colombia. Age, an aqueous flare, and a foreign body in the peripheral cornea were factors associated with keratitis.
